# Injury Risk in New Zealand Rugby Union: A Nationwide Study of Injury Insurance Claims from 2005 to 2017

**DOI:** 10.1007/s40279-019-01176-9

**Published:** 2019-09-11

**Authors:** Ken Quarrie, Simon Gianotti, Ian Murphy

**Affiliations:** 1 New Zealand Rugby, Wellington, New Zealand; 2 Accident Compensation Corporation, Wellington, New Zealand

## Abstract

**Objectives:**

The Accident Compensation Corporation is a compulsory, 24-h, no-fault personal injury insurance scheme in New Zealand. The purpose of this large-scale retrospective cohort study was to use Accident Compensation Corporation records to provide information about rugby injury epidemiology in New Zealand, with a focus on describing differences in risk by age and gender.

**Methods:**

A total of 635,657 rugby injury claims were made to the Accident Compensation Corporation for players aged 5–40 years over the period 2005–2017. Information about player numbers and estimates of player exposure was obtained from New Zealand Rugby, the administrative organisation for rugby in New Zealand.

**Results:**

Over three quarters of claims (76%) were for soft-tissue injuries, with 11% resulting from fractures or dislocations, 6.7% from lacerations, 3.1% from concussions and 2.0% from dental injuries. Body regions injured included shoulder (14%), knee (14%), wrist/hand (13%), neck/spine (13%), head/face (12%), leg (11%) and ankle (10%). The probability of a player making at least one injury claim in a season (expressed as a percentage) was calculated under the assumption that the incidence of claims follows a Poisson distribution. Players aged 5–6 years had a probability of making at least one claim per season of 1.0%, compared to 8.3% for players aged 7–12 years, 35% for age 13–17 years, 53% for age 18–20 years, 57% for age 21–30 years and 47% for age 31–40 years. The overall probability of making at least one claim per season across all age groups was 29%. The relative claim rate for adults (players aged 18 years and over) was 3.92 (90% confidence interval 3.90–3.94) times that of children. Ten percent of players were female, and they sustained 6% of the injuries. Overall, the relative claim rate for female players was 0.57 times that of male players (90% confidence interval 0.56–0.58). The relative claim rate of female to male players tended to increase with age. There were very few female players aged over 30 years; however, those who did play had higher claim rates than male players of the same age group (1.49; 90% confidence interval 1.45–1.53).

**Conclusions:**

Injuries resulting from rugby are distributed across the body, and most of the claims are for soft-tissue injuries. Rates of injury increase rapidly through the teenage years until the early 20 s; for male players they then decrease until the mid-30 s. For female players, the injury rate does not decrease as players move into their 30 s. Combining Accident Compensation Corporation injury claim data with national player registration data provides useful information about the risks faced by New Zealand’s community rugby players, and the insights derived are used in the development of rugby injury prevention programme content.

**Electronic supplementary material:**

The online version of this article (10.1007/s40279-019-01176-9) contains supplementary material, which is available to authorized users.

## Key Points

**Table:** 

This large-scale study provides significant new information about the injury epidemiology of community rugby players, and highlights differences in injury rates between genders and age groups.
Rates of rugby injury increase rapidly with age from childhood to adulthood; rates for adults (18 years of age and over) are about four times higher than rates for children (17 years of age and younger).
Injuries are distributed throughout the body; soft-tissue injuries comprise three quarters of injury claims.
In general, female players have lower rates of injury than male players.

## Introduction

Rugby union (rugby) is a field-based team sport characterised in part by the degree of physical contact players are permitted to use in contesting possession of the ball. According to World Rugby, which is the governing body of rugby union internationally, rugby union is played in 120 countries, with 8.5 million players participating in the sport [1].

Injury surveillance is fundamental to quantifying, and thus managing the risk of injury associated with a given activity [2–4]. The absence of information regarding injury risks means that participants cannot make informed decisions about whether to take part in the activity, and administrative or regulatory bodies cannot make evidence-supported decisions regarding risk mitigation strategies.

While there have been multiple studies of the epidemiology of rugby injuries from throughout the world, the preponderance of these have dealt with players at the elite level of the sport, who represent a small fraction of the overall playing population [5]. The injury epidemiology of players at the community level of the sport, and especially of female players, has been investigated in relatively few research studies [6–11]. Some researchers and safety advocates have suggested that injury surveillance in youth rugby is not as comprehensive as it ought to be, and the relative lack of research on the risks of injury for community-level players, especially for children, has led to competing claims about whether rugby is an acceptably safe sport [11–17].

With a few exceptions, most rugby-related injury surveillance projects published to date have involved the collection of injury information from researchers and/or medical personnel associated with teams. Another method of obtaining injury information across a defined population is via the analysis of injury insurance claims, as we describe below, and as King and colleagues have done for rugby league in New Zealand [18–20].

New Zealand, a country of approximately 4.8 million people in 2017 has had, since 1974, a 24-h no-fault levy- and taxpayer-funded injury insurance and rehabilitation scheme. The scheme was enacted by statute and is administered by the Accident Compensation Corporation (ACC). The ACC collects and stores information about all injuries in New Zealand that result in claims against the scheme, thus it provides a nationwide, all activity injury surveillance system. The ACC does not, however, monitor exposure to activities. New Zealand Rugby (NZR), which administers rugby union in New Zealand, records player numbers on an annual basis. The purpose of this paper is to use combined data from the ACC and NZR to describe the injury epidemiology and level of risk associated with participation in rugby across age groups and by gender in an entire country’s playing population.

## Methods

### Injuries and Player Numbers

The ACC obtains information about all rugby injuries that result in claims for medical assessment or treatment in New Zealand. The nationwide scope of the scheme means that, regardless of whether a player sustained an injury in school, club, amateur or professional rugby, the details of the injury are logged by the ACC. There are approximately 30,000 ACC-registered medical providers in New Zealand.

Because variations in injury definitions can lead to difficulties in comparing injury rates across studies, ‘consensus’ definitions for a number of sports, including rugby, have been developed [21]. Fuller and colleagues recommended that case definitions based on either ‘medical attention’ or ‘time-loss’ injuries should be used in studies of rugby injury epidemiology [21]. For the most part, ACC claims in New Zealand are synonymous with medical treatment for injuries (see www.acc.co.nz for details of the ACC system). Although it is technically possible for a rugby player in New Zealand to sustain an injury, obtain medical attention for it at the field of play and the medical treatment provider not to submit a claim to the ACC, in most cases, medical treatment injuries become ACC claims for all but very minor injuries.

Information was obtained from the ACC about 635,657 rugby injury claims for players aged 5–40 years that occurred from the 1 January, 2005 until the 31 December, 2017. Self-reported age and gender (recorded as male or female) are recorded for all ACC claims. Numbers of rugby players were obtained from the NZR player register, which also collects self-reported age and gender (again, as male or female).

While we also conducted analyses for each individual age, players were assigned to the following age groups: 5–6 years, 7–12 years, 13–17 years, 18–20 years, 21–30 years and 31–40 years for reporting purposes. In New Zealand, players aged 5 and 6 years play non-contact ‘tag’ rugby. Players aged 7–12 years (primary school age) play modified forms of rugby, with the contact elements of the sport introduced progressively over several years. Tackling usually begins for players at age 7 years. A description of the ‘rugby development model’ and the variations played by children of different ages is available online [22]. Players aged 13–17 years are of secondary school age. Players aged 18–20 years have normally left school; most play in age-graded competitions (e.g. Under 21 years). Players aged younger than 5 years (1.6% of all players), and older than 40 years (2% of all players) were excluded from the analyses.

We chose to report claim rates in two main ways; first, as the actual rate of claims per 1000 players by year, and second as the estimated rate of claims per 1000 player-hours of exposure to training and match play. The first was used because we were able to derive them directly from the ACC and NZR data. The second was used to facilitate comparisons with existing work because reporting injuries per 1000 player-hours has been the most commonly used convention in publications of rugby injury epidemiology [5, 11, 21].

### Injury Type and Body Region Definitions

Claims were classified into the following injury types:

- soft tissue (contusion, muscle strain, ligament strain);
- fracture or dislocation;
- cut/laceration;
- concussion/brain injury;
- dental injury; and
- ‘other’ (includes injuries to internal organs).

Body regions were grouped as follows, with the label for reporting in parentheses where it differs:

- head/face/ear/eye/nose (head/face);
- neck/spine;
- shoulder;
- arm/elbow (arm);
- wrist/hand/finger/thumb (wrist/hand);
- chest/abdomen/thorax (trunk);
- leg—excluding knee and ankle (leg);
- knee;
- ankle;
- foot/toe; and
- other—includes injuries to internal organs.

We excluded ‘multiple locations/unobtainable’ from the body region by injury type breakdown, as these accounted for less than 1% of all claims.

### Statistical Methods

The probability of a player making at least one injury claim in a season (expressed as a percentage) was calculated based on the method outlined by Parekh et al., under the assumption that the incidence of claims follows a Poisson distribution [23]. The probability of making at least one claim per year for any given player was:

$$100 \times \left( {1 - P\left( 0 \right)} \right),$$

where *P*(0), the probability of making zero claims, equals e^(−1*Injuries per player per year)^.

Note that ‘overall’ probabilities in the tables below are not linear sums of the component probabilities because of the non-linear transformation used to calculate Poisson probabilities.

Ninety percent confidence limits on rates and probabilities were calculated using Wilson’s method [24]. In the results below, where confidence intervals on rates are not presented, it is because the uncertainty in the estimate was negligible—the confidence limits range from the point estimate multiplied or divided (×/÷) by a factor of 1.01 through to ×/÷ 1.06. Comparisons of rates by gender, age group and injury site were calculated using the Genmod procedure in SAS (Version 9.4; SAS Institute, Cary, NC, USA).

### Supplementary Information

Because the volume of data analysed for this paper generated many more results than could be included in the traditional format of a scientific manuscript, two supplementary files have been produced as resources for researchers wishing to examine the data further or produce meta-analyses.

The details for estimating player exposure, and results presented for rates of claims per 1000 player-hours are provided in the Electronic Supplementary Material, together with a Microsoft Excel workbook (PC only; xlsx format) which includes a range of interactive charts and tables. Lists of relative rates (RR) are provided in the supplementary workbook as follows: between genders within age group by body region (SW sheet 11) and injury type (SW sheet 12); within genders between age groups by body region (SW sheet 13) and injury type (SW sheet 14).

## Results

**Fig. 1:**
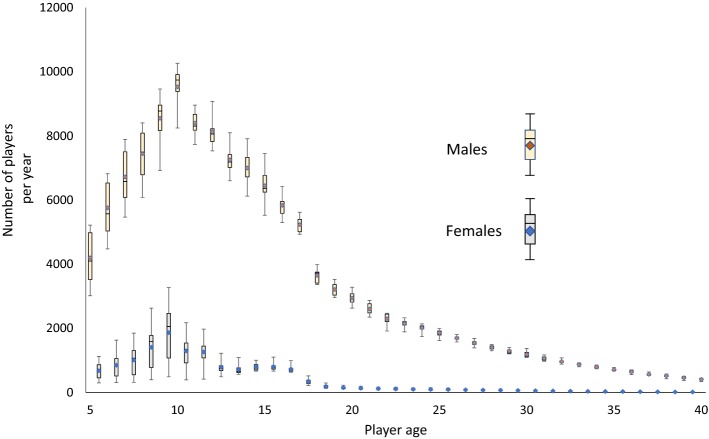
Box plots of average numbers of players per year by age and gender. The error bars represent the range of values. The mean is displayed by the coloured diamond in the box, and the median is the horizontal line within the box. The bottom of the box represents the first quartile, and the top of the box represents the third quartile

There was an average of 141,020 ± 7070 (mean ± standard deviation) players per year (125,900 ± 4160 male players; 15,120 ± 4370 female players). Approximately 6% of New Zealanders aged between 5 and 40 years are registered rugby players. The ages of 9 and 10 years have the greatest numbers of participating players, for both male and female players (Fig. 1).

### Claim Probabilities and Rates by Gender and Age

**Fig. 2:**
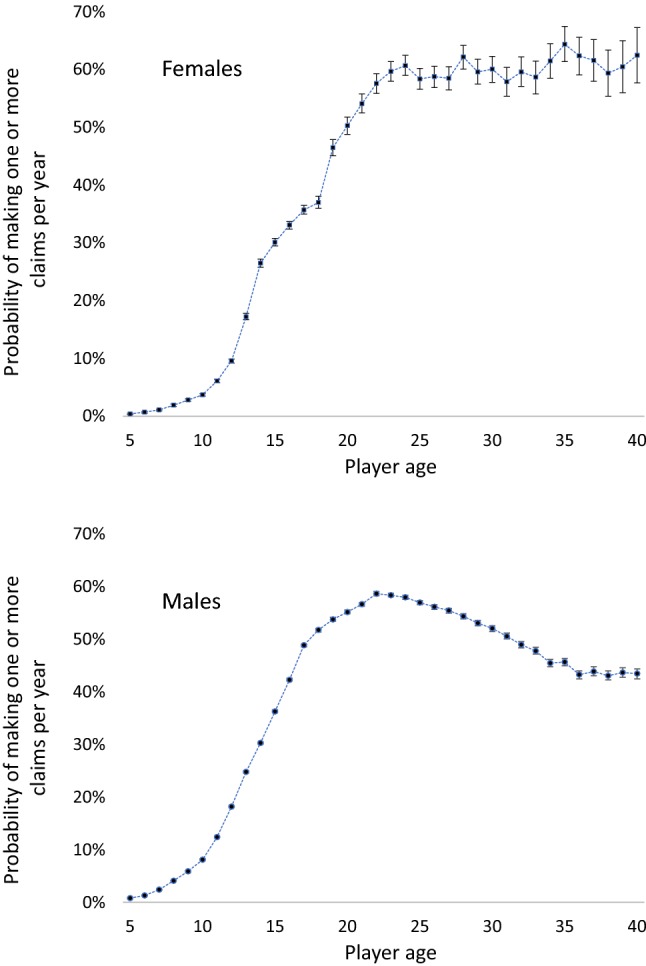
Probability of making at least one claim per year by age. Error bars represent 90% confidence intervals

For female players, the probability of making at least one injury claim increased from 0.4% per year at age 5 years through to between 58% and 64% for players aged 22 through to 40 years (Fig. 2). The largest differences in age-to-age claim probabilities were 8% from 12 to 13 years, 9% from 13 to 14 years and 10% from 18 to 19 years.

For male players, the probability of making an injury claim increased from 1% at age 5 years through to 52% at age 18 years, after which it peaked at about 58% during the early to mid-20 s and then decreased through to 44–51% for players aged 31–40 years. The largest differences in claim probabilities between any two ages were from age 12 to 13 years (7%) and from age 16 to 17 years (9%).

Children aged between 5 and 17 years inclusive made up 75% of the playing population and accounted for 42% of claims (Table 1). There was an average of one claim per year for every five players for those aged 5–17 years; the corresponding statistic was one claim for every 1.25 players for those aged 18–40 years.

**Table 1:** Accident Compensation Corporation claim and player numbers per year by gender and age group: 2005–17

Gender	Age group, years													
	5–6		7–12		13–17		18–20		21–30		31–40		Overall	
	Claims	Players	Claims	Players	Claims	Players	Claims	Players	Claims	Players	Claims	Players	Claims	Players
Female	7 ± 3	1267 ± 455^a^	364 ± 94	8103 ± 3086	1306 ± 409	3861 ± 638	384 ± 144	687 ± 178	801 ± 251	906 ± 200	275 ± 48	296 ± 52	3138 ± 925	15,120 ± 4370
Male	112 ± 22	10,148 ± 1519	4574 ± 661	49,233 ± 2555	14,186 ± 1597	31,676 ± 1855	7513 ± 762	9810 ± 585	15,012 ± 1130	18,039 ± 721	4362 ± 359	6995 ± 296	45,759 ± 4346	125,902 ± 4164
Total	119 ± 26	11,415 ± 1952	4938 ± 744	57,336 ± 5290	15,492 ± 1922	35,538 ± 1523	7899 ± 857	10,497 ± 502	15,813 ± 1282	18,945 ± 769	4637 ± 366	7291 ± 272	47,817 ± 5095	141,022 ± 7074
Percent of all claims for age group	0.2		10		32		16		20		9.5		100	
Percent of all players in age group		8		41		25		7		8		5		100

^a^Statistics are means per year ± one standard deviation

In most instances, players in older age groups had higher claim rates than those in younger age groups. The exceptions were for comparisons between both 18–20 years and 21–30 years with 31–40 years for the male players and both genders combined. Across both genders combined, rates of injuries to adults were 61–80 times higher than for those aged 5–6 years, 7.4–9.7 times higher than for those aged 7–12 years, and 1.46–1.92 times higher than for those aged 13–17 years.

Female players aged 31–40 years had the highest claim rate per 1000 players per year (930; Table 2); it was 174 (90% confidence interval (CI) 146–208) times higher than that of female players aged 5–6-years, 20.7 (90% CI 19.9–21.4) times higher than for female players aged 7–12-years, and two and three quarter times higher than female players aged 13–17 years (2.75; 90% CI 2.66–2.83). The rate for female players aged 13–17 years was five times higher than that players aged 7–12 years (5.23; 90% CI 5.17–5.28) and that for players aged 7–12 years was 8.40 (90% CI 7.04–10.05) times higher than that of players aged 5–6 years.

**Table 2:** Injury claim rates and claim probabilities by age group and gender

		Age group, years						
		5–6	7–12	13–17	18–20	21–30	31–40	Overall
Injury claims per 1000 players per year	Female	5.3^a^	45	338	559	884	930	169
	Male	10.8	93	448	766	832	623	276
	Both genders	10.4	86	436	752	835	636	265
Percent probability of making at least one claim per season	Female	0.53	4.4	29	43	59	61	19
	Male	1.1	8.9	36	54	57	46	31
	Both genders	0.95	8.3	35	53	57	47	29
Average number of seasons played per claim for players of that age group	Female	188	23	3.5	2.3	1.7	1.7	5.3
	Male	91	11	2.8	1.9	1.8	2.2	3.3
	Both genders	105	12	2.9	1.9	1.8	2.1	3.4

^a^90% confidence intervals for claim rates and claim probabilities are ≤ ×/÷ factors of 1.01

Male players aged 21–30 years had a claim rate per 1000 players per year of 832 (Table 2), which was a 76 (90% CI 72–79) times higher rate than male players aged 5–6 years; 8.96 (90% CI 8.89–9.03) times higher than male players aged 7–12 years and 1.86 (90% CI 1.85–1.87) times higher than male players aged 13–17 years. The rate for male players aged 13–17 years was almost five times higher than that for players aged 7–12 years (4.82; 90% CI 4.78–4.86), and that for players aged 7–12 years was 8.47 (90% CI 8.06–8.85) times higher than that of players aged 5–6 years.

### Gender

**Fig. 3:**
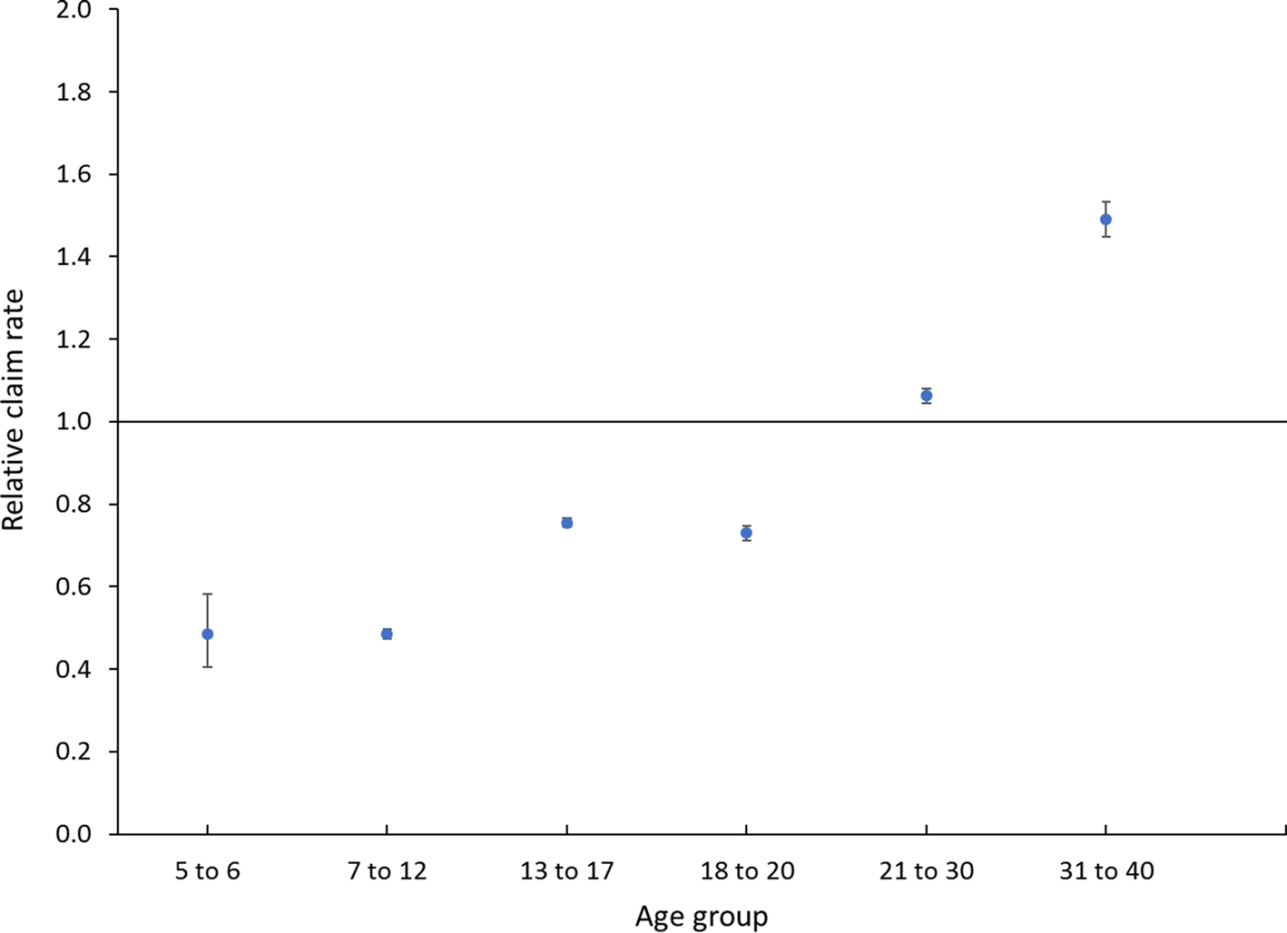
Relative female-to-male claim rates by age group. Error bars represent 90% confidence limits

Female players represented 9.5% of the playing population and made 5.8% of all claims. Only 12% of the female rugby players were adults, whereas 28% of male players were adults. Overall, the relative claim rate for female players was 0.57 times that of male players (90% CI 0.56–0.58). The relative claim rate of female to male players tended to increase with age (Fig. 3), and although there were very few female players aged over 30 years, those who did play had higher claim rates than male players of the same age group (1.49; 90% CI 1.45–1.53).

### Injury Types and Body Regions

**Fig. 4:**
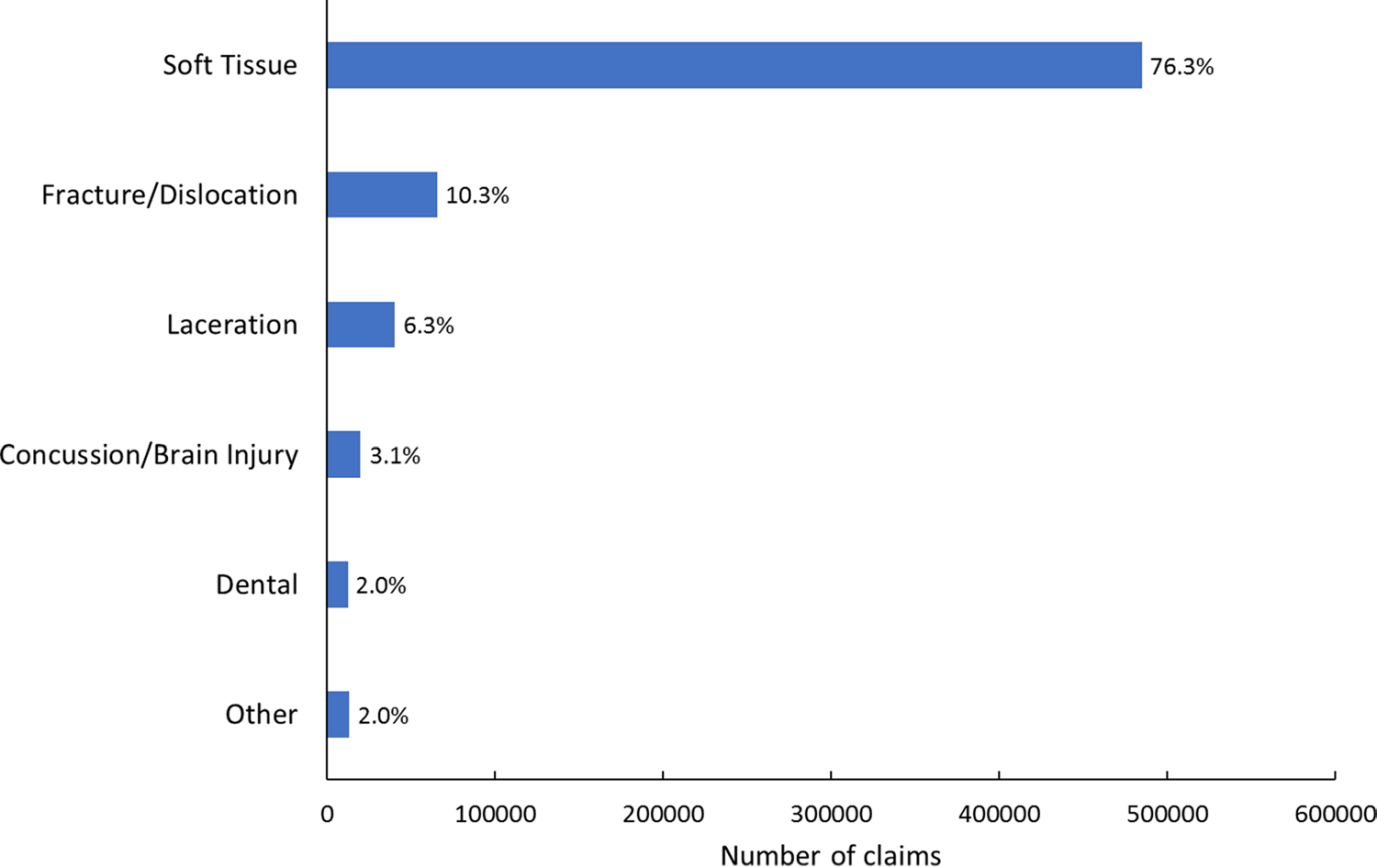
Number and percent of total rugby-related Accident Compensation Corporation claims by injury type, 2005–2017

**Fig. 5:**
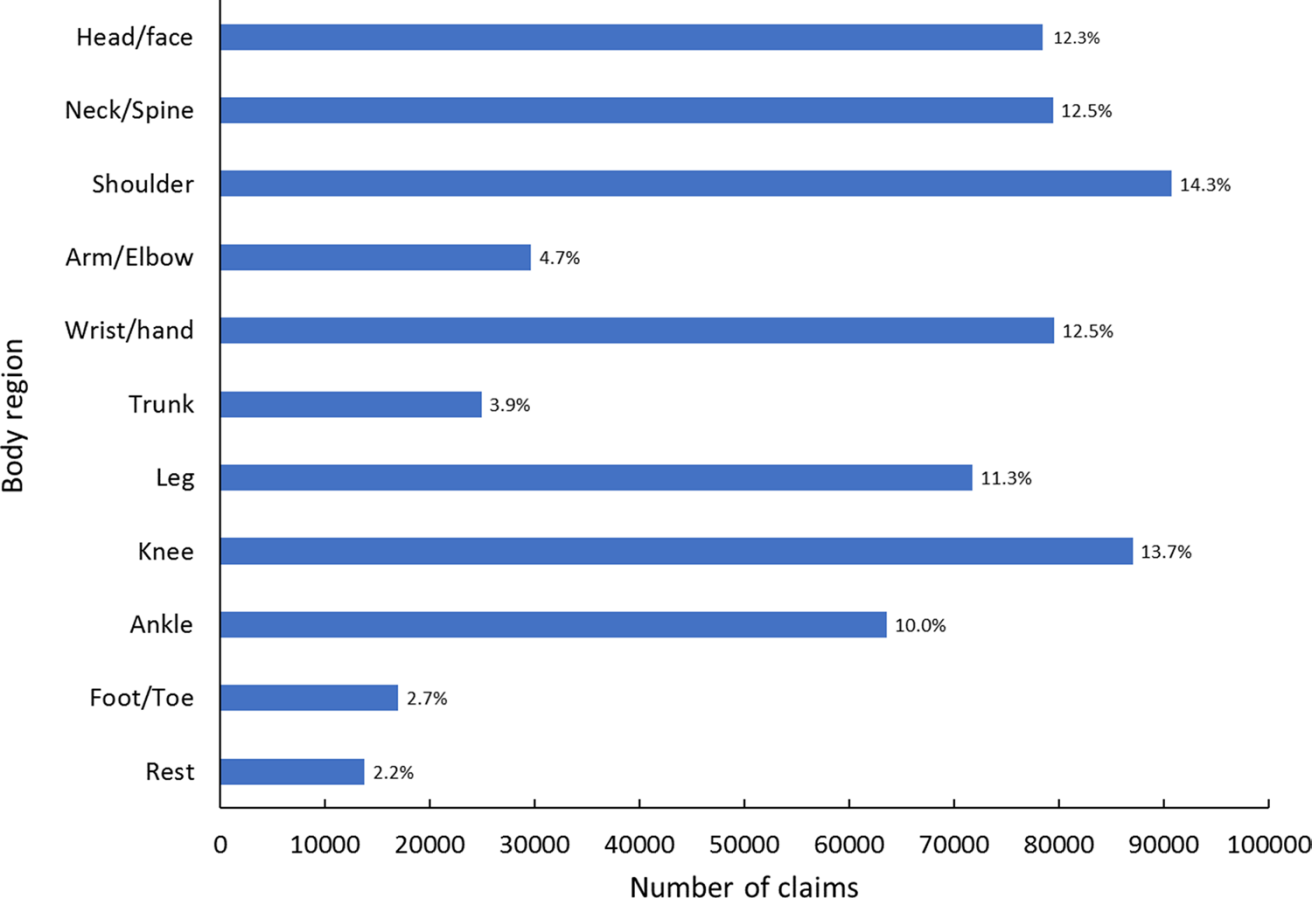
Number and percent of total rugby-related Accident Compensation Corporation claims by body region, 2005–2017

The number and percentage of claims by injury type are displayed in Fig. 4. Injuries were distributed across the body for all age groups (Fig. 5), with the head/face, neck/spine, wrist/hand, shoulder, knee, ankle and the remainder of the leg accounting for at least 10% of claims each.

Sixty-five percent of all claims (410,923 of 635,657) resulted from soft-tissue injuries to the following six body regions: neck/spine (12.4%), shoulder (11.8%), wrist/hand (8.4%), leg (10.0%), knee (12.6%) and ankle (9.5%). Eight percent of all claims, and 77% of fractures/dislocations resulted from fractures/dislocations to four body regions: the wrist/hand (3.6% of all claims; 35% of fractures/dislocations), shoulder (2.5%; 24%), arm (1.0%; 9%) and head/face (0.9%; 8%). The head/face was the body region that sustained the greatest number of cuts/lacerations (3.1% of all injuries; 49% of cuts/lacerations).

### Injury Type Differences Between Genders

Over the total playing population (players aged 5–40 years when considered as a group), male players had higher claim rates than female players for all injury types (Table 3; RR range 1.21–2.69). There were, however, age groups in which female players had higher claim rates than male players for specific injury types (Table 3). Female players aged 21–30 years had a rate of soft-tissue injuries that was 1.14 (90% CI 1.13–1.15) times higher than male players of the same age range (Table 3). Female players aged 31–40 years had higher rates of injury than male players of the same age range for all injury types (RR range 1.20–1.61) except lacerations (RR 0.58; 90% CI 0.49–0.69).

**Table 3:** Claim rates per 1000 players per year by gender, age group and injury type

Gender	Age group, years	Injury type						
		Soft tissue	Fracture/dislocation	Laceration	Concussion/brain injury	Dental	Other	Total
Female	5–6	3 (2.4–3.8)^a^	0.97 (0.64–1.5)	0.55 (0.32–0.95)	0.18 (0.07–0.47)	0.42 (0.23–0.79)	0.18 (0.07–0.47)	5.3 (4.5–6.4)
	7–12	32 (31–33)	8.5 (8.1–9)	1.8 (1.6–2)	1.0 (0.87–1.2)	0.86 (0.73–1)	0.85 (0.72–1.0)	44.9 (43.9–46.1)
	13–17	271 (267–275)	31 (30–33)	11 (9.8–11)	15 (14–16)	3.7 (3.3–4.2)	6.6 (6.1–7.3)	338 (334–343)
	18–20	464 (452–476)	45 (42–49)	16 (14-18)	18 (16–21)	7.4 (6–9)	8.2 (6.7–9.9)	559 (546–572)
	21–30	755 (742–768)	63 (59–67)	27 (25–30)	19 (17–21)	8.7 (7.4–10)	12 (11–14)	884 (870–898)
	31–40	796 (773–820)	69 (62–76)	24 (20–28)	9.6 (7.3–13)	13 (10–16)	18 (14–22)	930 (904–955)
	Mean	168 (167–170)	20 (19–20)	6.5 (6.2–6.8)	6.5 (6.2–6.8)	2.6 (2.4–2.8)	3.6 (3.4–3.9)	208 (206–209)
Male	5–6	5.7 (5.4–6.0)	1.8 (1.7–2.0)	2.0 (1.8–2.2)	0.24 (0.18–0.32)	0.84 (0.72–0.98)	0.42 (0.34–0.53)	11 (10.5–11.5)
	7–12	62 (62–63)	14 (14–14)	6.8 (6.6–6.9)	4.0 (3.9–4.1)	3.7 (3.6–3.9)	2.4 (2.3–2.5)	93 (92–94)
	13–17	327 (326–328)	53 (53–54)	27 (27–28)	21 (20–21)	10 (9.9–10)	9.6 (9.3–9.8)	448 (446–450)
	18–20	599 (596–603)	68 (67–69)	49 (48–50)	23 (22–24)	13 (13–14)	14 (13–14)	766 (762–770)
	21–30	662 (659–665)	71 (70–72)	55 (55–56)	17 (16–17)	11 (11–12)	15 (15–16)	832 (829–835)
	31–40	495 (491–499)	57 (56–59)	41 (40–42)	7 (6.6–7.5)	9.5 (9–10)	14 (14–15)	623 (619–628)
	Mean	276 (275–277)	38 (37–38)	24 (23–24)	11 (11–11)	7.3 (7.2–7.4)	7.4 (7.3–7.6)	363 (363–364)
Overall mean		264 (264–265)	36 (36–36)	22 (22–22)	10.8 (10.7–10.9)	6.8 (6.7–6.9)	7.0 (6.9–7.1)	347 (347–347)
Overall relative rate (male/female)		1.21 (1.16–2.26)	1.40 (1.30–1.51)	2.69 (2.43–2.99)	1.35 (1.13–1.61)	1.85 (1.63–2.10)	1.58 (1.33–1.89)	1.75 (1.74–1.77)

^a^Figures in parentheses are 90% confidence limits

Rates of laceration injuries to male players were higher than those to female players for each equivalent age group (Table 4; RR range 1.71–3.85). Male players also had higher rates of fractures/dislocation, dental and ‘other’ injuries than female players among players aged in the 7–12, 13–17, 18–20 and 21–30 years of age groups (RR range 1.13–4.32), and higher rates of soft tissue, and concussion/brain injury claims in the 7–12, 13–17 and 18–20 years of age groups (Table 4; RR range 1.21–3.93).

**Table 4:** Claim rates per 1000 players per year by gender, age group and body region

Gender	Age group, years	Body region										
		Head/face	Neck/spine	Shoulder	Arm	Wrist/hand	Trunk	Leg	Knee	Ankle	Foot/toe	Other
Female	5–6	1.3 (0.94–1.9)^a^	0.44 (0.19–0.99)	0.18 (0.07–0.47)	0.42 (0.23–0.79)	0.79 (0.5–1.2)	0.11 (0.02–0.56)	0.3 (0.15–0.63)	0.36 (0.19–0.71)	0.79 (0.5–1.2)	0.61 (0.36–1)	0.44 (0.19–0.99)
	7–12	4.5 (4.2–4.9)	2.8 (2.5–3)	2.9 (2.6–3.2)	3.6 (3.3–3.9)	13 (13–14)	0.87 (0.74–1)	2.2 (2–2.4)	4.5 (4.1–4.8)	5.5 (5.1–5.8)	3.9 (3.6–4.2)	0.97 (0.82–1.1)
	13–17	38 (37–39)	37 (36–39)	40 (39–42)	16 (15–17)	48 (47–50)	9 (8.4–9.7)	27 (26–29)	62 (60–64)	44 (42–45)	9 (8.3–9.7)	7.4 (6.8–8.1)
	18–20	57 (53–62)	71 (67–76)	73 (69–78)	20 (18–22)	60 (56–65)	16 (14–18)	56 (52–60)	107 (101–112)	76 (72–81)	13 (11–15)	9.5 (8–11)
	21–30	68 (64–72)	126 (121–132)	114 (109–120)	24 (22–27)	92 (88–97)	34 (32–37)	114 (109–119)	166 (160–172)	108 (103–113)	23 (20–25)	14 (12–16)
	31–40	55 (49–61)	159 (148–170)	105 (97–114)	34 (29–39)	94 (87–103)	41 (36–47)	128 (119–138)	159 (148–170)	112 (103–121)	24 (20–28)	20 (16–24)
	Mean	20 (19–21)	25 (24–26)	24 (23–25)	9.0 (8.7–9.4)	30 (29–31)	6.6 (6.3–6.9)	20 (19–21)	36 (35–37)	26 (25–27)	6.8 (6.5–7.1)	4.1 (3.8–4.3)
Male	5–6	2.9 (2.6–3.1)	0.77 (0.65–0.9)	0.78 (0.66–0.92)	1 (0.87–1.2)	1.4 (1.2–1.6)	0.25 (0.19–0.33)	0.79 (0.67–0.93)	1.1 (0.99–1.3)	0.7 (0.59–0.84)	0.81 (0.69–0.95)	0.5 (0.41–0.61)
	7–12	16 (16–16)	8.7 (8.5–8.8)	6.6 (6.4–6.7)	6.1 (6–6.3)	18 (18–19)	2.8 (2.7–2.9)	6.1 (6–6.3)	11 (11–11)	8.7 (8.5–8.9)	6.2 (6–6.4)	2.7 (2.6–2.8)
	13–17	66 (65–66)	57 (56–58)	66 (66–67)	23 (23–24)	65 (64–66)	14 (14–15)	41 (40–41)	56 (55–57)	37 (37–38)	12 (11–12)	10 (10–11)
	18–20	90 (88–91)	85 (84–87)	136 (134–138)	30 (29–31)	82 (81–83)	25 (25–26)	90 (88–91)	109 (107–110)	90 (89–91)	14 (13–15)	15 (14–15)
	21–30	86 (85–87)	110 (109-111)	128 (127–129)	34 (33–35)	82 (81–83)	38 (37–39)	114 (113–116)	119 (118–120)	89 (88–90)	16 (16–17)	16 (15–16)
	31–40	58 (57–59)	97 (95–98)	77 (76–79)	26 (25–27)	57 (56–58)	42 (41–43)	95 (94–97)	88 (86–90)	57 (55–58)	12 (12–13)	14 (14–15)
	Mean	45.5 (45.3–45.8)	45.5 (45.3–45.8)	52.5 (52.2–52.8)	17 (16.9–17.2)	45 (44.7–45.3)	14.5 (14.3–14.6)	41.4 (41.2–41.7)	48.8 (48.6–49.1)	35.7 (35.4–35.9)	9.5 (9.4–9.7)	7.9 (7.8–8)
Overall mean		42.8 (42.5–43.0)	43.3 (43.1–43.6)	49.5 (49.2–49.8)	16.2 (16.0–16.3)	43.4 (43.1–43.6)	13.6 (13.5–13.7)	39.1 (38.9–39.4)	47.5 (47.2–47.7)	34.7 (34.3–34.9)	9.3 (9.1–9.4)	7.5 (7.4–7.6)
Overall relative rate (male/female)		2.28 (2.22–2.34)	1.83 (1.79–1.87)	2.18 (2.12–2.23)	1.88 (1.81–1.96)	1.52 (1.48–1.55)	2.27 (2.17–2.39)	2.07 (2.01–2.12)	1.35 (1.32–1.38)	1.36 (1.32–1.39)	1.40 (1.34–1.47)	1.94 (1.82–2.06)

^a^Figures in parentheses are 90% confidence limits

### Injury Type Differences Across Age Groups

For both genders combined, the relative rate of all types of injury at least doubles as players move from the 7- to 12-year-old age group to teenage and adult age groups (RR range 1.99–11.5; SW sheet 14). With the exceptions of concussion/brain injuries, for which rates were higher for players aged 13–17 years, and dental claims, for which there was little difference, claim rates for adults were higher than for the 13—17-year old age group. Soft-tissue injury claim rates were 8.78–11.5 times higher for players aged 18–40 years than for those aged 7–12 years, and 1.58–2.08 times higher for those aged 18–40 years than for players aged 13–17 years. Players aged 13–17 years had a rate of soft-tissue injuries 5.56 times that of those aged 7–12 years.

### Body Region Differences Between Genders

Male players aged 7–12, 13–17, 18–20 and 21–30 years had higher claim rates than female players of the equivalent age group for head/face, shoulder, arm, trunk and leg and ‘other’ injuries (Table 4; RR range 1.11–3.53). Male players also had higher claim rates than female players for the following age group by body region combinations: neck/spine, wrist/hand and foot/toe injuries for 7–12, 13–17 and 18–20 years (RR 1.10–3.13), and finally, knee (RR 2.41; 90% CI 2.23–2.61), and ankle injuries for 7–12 years (RR 1.59; 90% CI 1.48–1.71) and ankle injuries for 18–20 years (RR 1.18; 90% CI 1.11–1.26). Male players had lower rates than female players for knee and ankle injuries in the 13–17, 21–30 and 31–40 year age groups, for neck/spine, wrist/hand and foot/toe injuries for the 21–30 and 31–40 year age groups, and additionally for shoulder, arm, leg and ‘other’ injuries for the 31–40 year age group (Table 4; RR 0.51–0.90).

### Body Region Differences Across Age Groups

When the genders were grouped, rates of claims across the entire body were substantially higher for age 13–17 years than age 7–12 years (RR range 1.92–10.5). The shoulder was the body region with the highest relative rate between the players aged 7–12 and 13–17 years. Relative injury rates for the following body regions increased by age group from 7–12 years through to 21–30 years, and then decreased in the 31–40 year age group: neck/spine, arm, wrist/hand, trunk, leg, knees and foot/toe. Whereas there was a decrease in claim rates for all body regions except the trunk for male players aged 31–40 years when compared with male players aged 21–30 years, female players aged 31–40 years had higher rates than the other age groups for the trunk, neck/spine, arm, leg and ‘other’ body regions.

## Discussion

The results presented here provide significant new knowledge about the injury experience of community-level rugby players for two main reasons. First, the results represent rugby injuries from an entire country’s playing population, and they have been collected over a 13-year period. Because the sample is so large, there is less sampling uncertainty in the rates presented than has been the case for previous studies of rugby injury epidemiology. Second, injury rates and probabilities have been presented for community-level female and male players across a wider range of ages than has been presented to date (although we limited it to the 96.4% of players who were aged between 5 and 40 years). The ACC is unique in providing a nationwide all-cause injury surveillance system in a country that has rugby as a major sport. New Zealand Rugby is also unusual among sporting organisations in New Zealand in that it maintains a comprehensive annually updated database of player registrations. The combination of data from the two organisations enables rates of injury claims per player-year to be calculated with reasonable accuracy.

### Study Limitations

A limitation of our study is that the proximal cause of injury within rugby is not systematically collected by the ACC (i.e. whether the injury occurred in a tackle, ruck, scrum), although a text description of the event causing the injury is completed. Beyond that, the structure of the ACC system itself could impact on the accuracy of data collection, especially with respect to injury type. On registering a claim, often prior to a detailed assessment and/or investigation of the injury, health providers are required to nominate a diagnosis. This diagnosis is then lodged with other details of the claim at the beginning of the injury management process. In most cases, no allowance is made for the possible revision of this diagnosis after the injury episode to a more accurate diagnosis once more information about the injury becomes available. The accuracy of this process is therefore influenced by different healthcare discipline backgrounds and individual provider experience. Further, the fact that certain types of claims can only be made by specific groups of health providers and that ACC has a fee-for-service funding model may influence claim lodgement behaviour. Unfortunately, we are unable to quantify how much of an effect these factors may have on claim statistics but our belief is that for the relatively gross body region and injury type groupings we have used, any such miscoding would be unlikely to have had a major impact on population-level statistics. Although we are not aware of any regulatory changes to the scheme over the study period that would have materially altered claim rates or patterns, we believe there has, however, been an increase in service provision, which is likely to have resulted in a greater number of soft-tissue injury claims over time.

### Injury Epidemiology

There were large variations in injury claim rates by age. Rates of injury for players under the age of 13 years are much lower than those of older players, and the injury risks for 5- and 6-year-old players are negligible. There are increases in claim rates and probabilities throughout the teenage years. The increase in injuries may be a function of players becoming bigger and faster, and therefore able to generate greater energy in collisions, as they mature. The increase in injury rates by age we have presented here is consistent with most previous reports of the epidemiology of youth rugby [25]. For male players, the biggest differences in claim rates between any consecutive ages were observed for players between the ages of 12 and 13 years (which coincides with the move from primary to secondary school) and for players between the ages of 16 and 17 years. Players aged 17 years are those most likely to be involved in ‘First XV’ rugby, which, in New Zealand, is a particularly competitive environment. For female players, there was a large difference between the ages of 12 and 13 years, and another between the ages of 18 and 19 years, which presumably represents the age at which players enrol in adult, rather than school, rugby competitions.

Although there has been a significant increase in the number of female players in New Zealand over the past 13 years, male players still account for 90.5% of all players, and 94% of all injury claims. A finding that we did not expect was that the rate of injuries to adult female players aged 21–30 years was similar to that of male players, and that of female players aged 31–40 years was substantially higher than that of male players of the same age. While further research is needed to ascertain the reasons for this finding, a possible explanation is that there are fewer teams available for female players than male players, which forces the female players who wish to continue to play to participate in teams that often include international and provincial representatives and are thus highly competitive. To the best of our knowledge, there are at present no social grades available in which older female players can participate. By contrast, as male players move into their 30 s, they are more able to opt to play in social teams.

Issues of ‘matching’ players against opponents of similar size and ability, and the effect such matching may have on injury rates also exist within teenage grades [26]. Among teenage female players, there is usually only a single competition or ‘grade’ available for participation, which means that players of very different sizes and maturity levels can be competing against each other. For male players in urban areas, there are more likely to be options based on combinations of age and weight; however, in rural areas there are not usually enough players to permit more than a single grade for players of a given age. Further examination of the influence of geographic location and competition structures on injury risk and participation would be useful for risk management purposes.

By far the most common injury claim type in New Zealand rugby is for soft-tissue injuries (76%), and almost one third of adult players can expect to make at least one claim for a soft-tissue injury from rugby per year. Although other injury claim types, such as fractures and dislocations, lacerations, concussions and dental injuries, occur at lower rates than soft-tissue injuries, they are nevertheless important to understand for prevention purposes because typical injury severity and claim costs differ across the injury types.

Consistent with previous rugby injury surveillance reports are the findings that injuries are distributed across the body, and that the shoulder, neck/spine and lower limb are body regions that have a relatively high incidence of soft-tissue injuries [25]. The shoulder, lower arm/hand and knee are body sites that have relatively high rates of fracture/dislocation, although the probability per player of making a claim for any of these injuries is less than 3% per year across any of the age groups. The authors examine the ACC claim data on a regular basis. Despite this, a finding that surprised us was the fact that, with respect to fractures, claims for injuries to the wrist, hand, fingers and thumb were the most common.

The rate of laceration injuries [27] is much higher among male players than among female players (over 2.7 times as high when compared across all age groups), which may indicate differences between the genders in the perceived acceptability of intentional acts of illegal play such as striking, kicking or trampling on opponents, and/or a more aggressive approach in going into contact situations. Although male players had higher rates of injuries to all body regions when considered across all age groups, the differences were greater for injuries to the shoulder and head/face and smaller for injuries to the knee and ankle. For players aged over 20 years, female players had higher rates of neck/spine, wrist/hand, knee, ankle and foot/toe injuries than did male players.

### Concussion/Brain Injury

Issues regarding difficulties in establishing reliable rates of concussion in the absence of consistently applied case definitions have been highlighted previously [28–30]. The rate of concussion claims made to the ACC almost certainly reflects only a small subset of the number of concussions players sustain, based on the range of rates derived from previous studies of rugby injury epidemiology [31–33].

Reasons for players not seeking treatment for concussions (and thus not making a claim) may include lack of recognition of the injury by the injured player or their family or teammates, or a belief that the injury is of insufficient severity to require presentation to medical providers [34–36]. For injuries other than concussion, players can return to play when they or their guardians see fit, either in conjunction with or independent of medical advice. The requirement that community-level rugby players who are diagnosed with a concussion must undergo a return to play protocol, which takes players at senior level at least 21 days to complete, and those at under 18 level at least 23 days, may be perceived by some players as a disincentive for disclosing that they have sustained a concussion [37, 38]. Further research into the extent to which concussions are underreported in New Zealand rugby, and the knowledge, attitudes and behaviours of rugby participants with respect to concussions, is warranted.

### Accident Compensation Corporation and NZ Rugby Injury Surveillance and Injury Prevention

The injury information provided by the ACC system combined with information about player numbers and exposure from NZR should help address some of the concerns raised recently about the lack of knowledge of the risk of childhood rugby injuries [11, 13, 16]. Little research regarding the relative risks of other sports compared to rugby in New Zealand has been completed, partly because there is a lack of reliable denominator data (participant numbers; participant exposure) available for most other sports. Ideally, an adjunct injury surveillance system would operate in New Zealand to complement the information provided by the ACC. Such a system would provide details about participant exposure across activities and capture a greater depth of information about the inciting events and mechanisms of injury than is currently available via the ACC. An example of the implementation and utility of such a system within New Zealand rugby has previously been published [9].

Previous research by King and colleagues using the ACC to describe injury patterns in rugby league and rugby union has provided useful information about the total numbers and costs of claims [18, 20, 39, 40]. The current work, by including player numbers to provide a ‘denominator’, adds value to the earlier publications by showing, as well as the numbers of injuries sustained, relative rates by age group and gender. In doing so, our work is similar in approach to recently published research on risks in team sports from Sweden [41, 42]. As was the case in our study, Åman et al. used a comprehensive nationwide injury insurance database combined with player numbers obtained from the registration of players. Across four team sports (floorball, football [soccer], handball and ice hockey), knee injuries were found to be the most common injuries, and injuries to the knee, head/face and upper limb (including the shoulder) were identified as body regions upon which injury prevention interventions should focus.

Based on our findings, we endorse the recommendations of Åman et al. Previous studies have found that rugby injuries are primarily the result of contact between players, especially tackles [5, 43]. RugbySmart is a nationwide rugby injury prevention partnership programme between ACC and NZR, and compulsory injury prevention sessions have been delivered to coaches and referees under its banner since 2001. Much of the advice in RugbySmart has centred on physically preparing players for the demands of the sport given their level of play, how to most safely and effectively develop the skills required in the contact elements of the sport, and how to manage injuries when they do occur. Advising participants about recognising and managing concussions is an important element of RugbySmart. The results of our current work are already being incorporated into the content of RugbySmart for 2020 and beyond.

## Conclusions

This large-scale retrospective cohort study provides information about injury claim rates and probabilities across an entire rugby playing population from 2005 to 2017. There were considerable variations in injury claim rates by age, with children being at a substantially lower risk than adult players. Overall, male players have higher claim rates than female players, although the differences between the genders vary with age. Injuries are distributed across the body, and most of the claims are for soft-tissue injuries. Rates of injury increase rapidly through the teenage years; for male players, they then level off through the early 20 s and decrease for players in their 30 s. For female players, the injury rate does not decrease as players move into their 30 s, but there are few female players who continue to participate beyond their 20 s.

The combination of ACC injury claim data with national player registration data provides a useful approach to understanding the risks faced by New Zealand’s community rugby players. The information derived helps inform the development of future injury prevention materials for the RugbySmart programme, and is in accordance with recognised public health models of preventing injuries in sport [2–4].

## Electronic supplementary material

Below is the link to the electronic supplementary material.

Supplementary material 1 (PDF 204 kb)

Supplementary material 2 (XLSX 379 kb)
