# Correction to: Injury Risk in New Zealand Rugby Union: A Nationwide Study of Injury Insurance Claims from 2005 to 2017

**DOI:** 10.1007/s40279-019-01202-w

**Published:** 2019-10-09

**Authors:** Ken Quarrie, Simon Gianotti, Ian Murphy

**Affiliations:** 1 New Zealand Rugby, Wellington, New Zealand; 2 Accident Compensation Corporation, Wellington, New Zealand

## Correction to: Sports Medicine 10.1007/s40279-019-01176-9

Page 11, column 2, section 4.2 Injury Epidemiology, paragraph 4:

The following sentence, which previously read:

By far the most common injury claim type in New Zealand rugby is for soft-tissue injuries (76%), and almost one third of adult players can expect to make at least one claim for a soft-tissue injury from rugby per year.

should read:

By far the most common injury claim type in New Zealand rugby is for soft-tissue injuries (76%), and about 43% of adult players can expect to make at least one claim for a soft-tissue injury from rugby per year.

