# Supplementary material for: Injury Risk in New Zealand Rugby Union: A Nationwide Study of Injury Insurance Claims from 2005 to 2017
Source: Sports Med. 2019 Sep 11;50(2):415–28. doi: 10.1007/s40279-019-01176-9 (PMC6985044; doi:10.1007/s40279-019-01176-9)
Supplement: Supplementary file 1 — Supplementary material 1 (PDF 204 kb) [file 40279_2019_1176_MOESM1_ESM.pdf]

Injury risk in New Zealand rugby union. A nationwide study of injury insurance claims from 2005 to 2017

Journal: Sports Medicine
Authors: Ken Quarrie, Simon Gianotti and Ian Murphy
Corresponding author: Ken Quarrie, Chief Scientist, New Zealand Rugby
Email: ken.quarrie@nzrugby.co.nz

Supplementary File 1.

1. Estimated exposure

Because we were unable to directly measure exposure for each player, we developed estimates of exposure for players at each age group. The exposure estimates were developed to facilitate comparisons with existing work, because reporting injuries per 1000 player-hours has been the most commonly used convention in publications of rugby injury epidemiology [1-3].

The estimates were generated from information about competition draws provided by NZR provincial union staff, and combined with data obtained from previous surveys of rugby player exposure and injuries in New Zealand community and schools rugby [4-6]. The following assumptions about exposure were made:

- Match exposure as a percentage of all rugby exposure ranged between 20% and 30% depending on age-group.
- Players aged under 13 and over 30 typically have lower exposure to training than players in their teens and twenties.
- Overall, players participated in approximately 2/3 of the match time available (because of substitutes and injuries).
- Exposure at each age group represented the average over the study period.

We recognise that there is substantial inter-grade (and inter-player) variability in exposure. “Social” teams tend to spend relatively less time practicing, and often have fewer matches, than teams in more competitive grades. Depending on whether players are selected into representative (regional and/or national) teams, their exposure can be significantly greater than average; conversely, some players may only appear in a single match or training session, or part of a match or training session, in a given year. There is also a degree of exposure for people who play rugby on a casual basis, but do not register as players with a team. While these players probably account for only a small amount of the total exposure, their exclusion from the NZR player registration system means that the rates of injury presented below are higher than would be the case than if they were included. Details about exposure estimates by age group are provided in Table 1.

Table 1. Estimated player exposure per year by age group

	Age group					
	5–6	7–12	13–17	18–20	21–30	31–40
Average number of matches played	16	16	16	19	20	18
Average match minutes available per player	650	891	1137	1394	1568	1425
Average match minutes played per player (minutes available * 2/3)	433	594	758	920	1045	950
Average match hours per player	7.2	9.9	12.6	15.3	17.4	15.8
Average practice hours per player	16.6	25	52	58	57	36.5
Total exposure (hours per season)	23.8	34.9	64.6	73.3	73.4	52.3

ACC claims do not require those completing the claim forms to specify whether the injury was sustained during training/practice or matches. Previous surveys of injuries among samples of rugby players in school and community level teams have indicated that the relative percentage of match injuries to training/practice injuries is typically in the range of 80-90%,[4, 7] whereas at professional level the

percentage of match injuries is around 65% [8]. Following an examination of the proportion of injury claims lodged on each day of the week, and taking into account the previous studies, we have assumed that 75% of claims resulted from matches, and have reported estimates of what the match and training claim rates were on that basis.

2. Results

Table 2. Estimated Injury claim rates per 1000 player-hours by age group and gender

		Age group [a]						
		5-6	7-12	13-17	18-20	21-30	31-40	Overall
Injury claims per 1000 player match-hours	Females	0.56	3.4	20	27	38	44	14
	Males	1.1	7.0	27	38	36	29	22
	Both genders	1.1	7.0	26	37	36	30	21
Injury claims per 1000 player training-hours	Females	0.08	0.45	1.6	2.4	3.9	6.4	1.5
	Males	0.17	0.93	2.2	3.3	3.7	4.3	2.3
	Both genders	0.16	0.86	2.1	3.2	3.7	4.4	2.2
Injury claims per 1000 player hours (matches and training combined)	Females	0.22	1.3	5.2	7.6	11.4	17.8	4.5
	Males	0.46	2.7	6.9	10.4	10.8	11.9	7.1
	Both genders	0.44	2.5	6.7	10.3	10.8	12.2	7.0

[a] 90% CIs for claim rates and claim likelihoods range from x/÷ factors of ≤1.07 for matches and overall and ≤1.16 for training for 5-6-year-olds. All others are ≤ x/÷ factors of 1.01 for matches and overall and ≤ x/÷ 1.02 for training.

3. Discussion

Because of the range of assumptions that were required to produce estimates of exposure, the results in Table 2 should be considered 'indicative', rather than 'definitive' of the injury rates per 1000 player hours of exposure to training and matches. Under the assumption that 75% of injuries occurred during matches, match and training claim rates are consistent with those reported in previous studies of injury rates among community-level rugby players, the different injury definitions used notwithstanding. For example, the estimated match injury rate for male 13-17 year olds in the current study was 27 injuries per 1000 player hours, which is essentially identical to the rate of 26.7 per 1000 player hours using an 'all injury' definition reported for youth players aged 12 to 18 derived from a meta-analysis [1]. Studies of youth players from Australia (players aged 10 to 17) and Ireland published subsequent to the meta-analysis reported rates of 24 injuries and 29 injuries per 1000 player match-hours respectively [9, 10].

A meta-analysis of injury rates in male amateur rugby reported a pooled estimate of 46.8 (95% CI 34.4 to 59.2) match injuries per 1000 player hours [11], which is somewhat higher than our match injury estimates for 18-20, 21-30 and 31-40 year old males of 37, 36 and 30 injuries per 1000 player hours respectively. The authors of the meta-analysis noted the low number of studies (six) available for meta-analysis, along with differences in study design, data collection and reporting methods as limitations; the total number of injuries used in the meta-analysis was 2340 [11]. One of the advantages of using the ACC system in our study was that the number of injuries available for analysis was greater than that published in all previous studies of community rugby combined (i.e. 349539 injuries for males aged 18 and over).

4. References

1. Freitag A, Kirkwood G, Scharer S, et al. Systematic review of rugby injuries in children and adolescents under 21 years. *Br J Sports Med* 2015;49(8):511-9.
2. Fuller CW, Molloy MG, Bagate C, et al. Consensus statement on injury definitions and data collection procedures for studies of injuries in rugby union. *Br J Sports Med* 2007;41(5):328-31.
3. Williams S, Trewartha G, Kemp S, et al. A meta-analysis of injuries in senior men's professional Rugby Union. *Sports Med* 2013;43(10):1043-55.
4. Bird YN, Waller AE, Marshall SW, et al. The New Zealand Rugby Injury and Performance Project: V. Epidemiology of a season of rugby injury. *Br J Sports Med* 1998;32(4):319-25.
5. Chalmers DJ, Samaranayaka A, Gulliver P, et al. Risk factors for injury in rugby union football in New Zealand: a cohort study. *Br J Sports Med* 2012;46(2):95-102.
6. Durie R, Munroe A. A prospective survey of injuries in a New Zealand schoolboy rugby population. *New Zealand Journal of Sports Medicine* 2000;28(4):84-91.
7. Brooks JH, Kemp SP. Recent trends in rugby union injuries. *Clin Sports Med* 2008;27(1):51-73.
8. Kemp S, S. W, Brooks JH, et al. England Professional Rugby Injury Surveillance Project, 2016-2017 season report, 2018.
9. Leung FT, Franettovich Smith MM, Brown M, et al. Epidemiology of injuries in Australian school level rugby union. *J Sci Med Sport* 2017;20(8):740-744.
10. Archbold HA, Rankin AT, Webb M, et al. RISUS study: Rugby Injury Surveillance in Ulster Schools. *Br J Sports Med* 2017;51(7):600-606.
11. Yeomans C, Kenny IC, Cahalan R, et al. The incidence of injury in amateur male rugby union: a systematic review and meta-analysis. *Sports Med* 2018;48(4):837-848.
